# Single cocaine exposure attenuates the intrinsic excitability of CRH neurons in the ventral BNST via Sigma-1 receptors

**DOI:** 10.1515/tnsci-2022-0339

**Published:** 2024-04-24

**Authors:** Jintao Wu, Yue Zhao

**Affiliations:** School of Brain Science and Brain Medicine, Zhejiang University, Hangzhou, China; School of Basic Medical Sciences, Hangzhou Normal University, Hangzhou, China; Liangzhu Laboratory, Zhejiang University Medical Center, MOE Frontier Science Center for Brain Science and Brain-Machine Integration, State Key Laboratory of Brain-Machine Intelligence, Zhejiang University, Hangzhou, China

**Keywords:** cocaine, CRH neurons, ventral BNST, intrinsic excitability, Sigma-1 receptors

## Abstract

The ventral bed nucleus of the stria terminalis (vBNST) plays a key role in cocaine addiction, especially relapse. However, the direct effects of cocaine on corticotropin-releasing hormone (CRH) neurons in the vBNST remain unclear. Here, we identify that cocaine exposure can remarkably attenuate the intrinsic excitability of CRH neurons in the vBNST *in vitro*. Accumulating studies reveal the crucial role of Sigma-1 receptors (Sig-1Rs) in modulating cocaine addiction. However, to the authors’ best knowledge no investigations have explored the role of Sig-1Rs in the vBNST, let alone CRH neurons. Given that cocaine acts as a type of Sig-1Rs agonist, and the dramatic role of Sig-1Rs played in intrinsic excitability of neurons as well as cocaine addiction, we employ BD1063 a canonical Sig-1Rs antagonist to block the effects of cocaine, and significantly recover the excitability of CRH neurons. Together, we suggest that cocaine exposure leads to the firing rate depression of CRH neurons in the vBNST via binding to Sig-1Rs.

## 1 Introduction

As a key part of the extended amygdala, the bed nucleus of the stria terminalis (BNST) plays crucial roles in anxiety and addiction [1,2]. A collection of nuclei constitute such complex structure, and the anterior BNST nuclei can be divided into the dorsal and ventral parts by the anterior commissure [3]. Previous studies demonstrate that ventral BNST (vBNST) is associated with stress-induced cocaine seeking and can manipulate dopamine neurons in the ventral tegmental area (VTA) [4]. Considering the essential role of VTA in cocaine addiction, it is not surprising that projections from vBNST to VTA implicate in the expression of cocaine preference [5]. Moreover, also through its VTA projections, vBNST serves as a prominent node for modulating motivation, as well as anxiety [6]. Besides, vBNST is activated during unpredictable threats or exposure to stressors, especially for its corticotropin-releasing hormone (CRH) neurons [7,8].

Stressors elicit the synthesis and release of CRH, which is central for the classic stress axis [9]. It is well documented that the recruitment of brain stress system drives drug seeking, and contributes to the “dark side” of addiction [10]. Notably, previous investigations suggest that CRH neurons in the vBNST innervate VTA. Therefore, CRH neurons in the vBNST should be critical for the process of cocaine addiction, particularly stress-induced cocaine relapse [11]. Strikingly, a seminal study shows that a single cocaine exposure initiates long-term potentiation in VTA dopamine neurons [12]. Given the relation of CRH neurons in the vBNST and dopamine neurons in the VTA as well as their essential roles in cocaine addiction, we propose that a single exposure of cocaine may also cause the alteration of intrinsic plasticity in CRH neurons of the vBNST.

Emerging evidence indicate that Sigma-1 receptors (Sig-1Rs) play key roles in cocaine addiction, affecting the intrinsic excitability of neurons via regulating a plethora of ion channels [13]. Moreover, cocaine *per se* is a well-known Sig-1Rs agonist [14,15]. Cocaine could diffuse through the plasma membrane and interact with Sig-1Rs intracellularly [16]. Here, we found that cocaine exposure could dramatically reduce the firing rate of CRH neurons in the vBNST. Interestingly, our results are similar to the reports of medium spiny neurons in the nucleus accumbens shell, which uncover Sig-1Rs as the underlying effectors [17,18]. Thus, we proposed that the effect of cocaine on CRH neurons might also exert via Sig-1Rs. Our investigation demonstrated that Sig-1Rs antagonist BD1063 could rescue the firing rate depression in CRH neurons caused by cocaine exposure, indicating Sig-1Rs as potential modulators of intrinsic excitability in the CRH neurons of vBNST.

## 2 Materials and methods

### 2.1 Animals

To visualize CRH neurons in the vBNST, we crossed CRH-ires-Cre mice (Jackson Laboratory, Stock No.012704) with Ai14 (Jackson Laboratory, Stock No. 007914) reporter mice. The CRH-ires-Cre-Ai14 male mice (considering the sexual dimorphic property of BNST [19], only male mice were used in our investigation) were housed in groups of 3–4 and kept in a temperature-controlled environment with 12 h dark/12 h light cycle.

### 2.2 Reagents

Cocaine hydrochloride was obtained from Qinghai Pharmaceutical Factory (Qinghai, China) and dissolved in 0.9% NaCl. Tetrodotoxin (ChemFaces, China), kynurenic acid (Sigma-Aldrich), and BD1063 (Abcam) were dissolved in water. Picrotoxin (Sigma-Aldrich) was dissolved in DMSO. All the chemicals used for the preparation of cutting solution, incubation solution, and recording solution were purchased from Sigma-Aldrich.

### 2.3 Electrophysiology

Brain slices containing the vBNST were prepared from 8 to 20 weeks CRH-ires-Cre-Ai14 male mice. Mice were anaesthetized with isoflurane and then decapitated. The brains were quickly harvested, and sliced coronally (300 μm) in ice-cold N-methyl-D-glucamine (NMDG) solution (in mM: 92 NMDG, 2.5 KCl, 1.25 NaH_2_PO_4_, 30 NaHCO_3_, 20 HEPES, 25 glucose, 2 thiourea, 5 Na-ascorbate, 3 Na-pyruvate, 0.5 CaCl_2_·2H_2_O, and 10 MgSO_4_·7H_2_O) [20]. Slices were incubated for about 13 min in the NMDG solution at 33°C. After that, slices were transferred into the 4-(2-Hydroxyethyl)-1-piperazine ethanesulfonic acid (HEPES) solution (in mM: 92 NaCl, 2.5 KCl, 1.25 NaH_2_PO_4_, 30 NaHCO_3_, 20 HEPES, 25 glucose, 2 thiourea, 5 Na-ascorbate, 3 Na-pyruvate, 2 CaCl_2_·2H_2_O, and 2 MgSO_4_·7H_2_O) and kept at room temperature for about 1 h. Then, slices were transferred into new beakers with HEPES solution and treated with cocaine (3 μM) or saline (equal volume), or pretreated with BD1063 (500 nM) for 30 min. Finally, slices were transferred into the recording chamber and superfused with 2–4 mL min^−1^ artificial cerebrospinal fluid (ACSF) (in mM: 124 NaCl, 2.5 KCl, 1.25 NaH_2_PO_4_, 24 NaHCO_3_, 12.5 glucose, 5 HEPES, 2 CaCl_2_·2H_2_O, and 2 MgSO_4_·7H_2_O) mixed with 2 mM kynurenic acid and 100 μM picrotoxin to block glutamate receptors and GABA_A_ receptors, respectively, the temperature was monitored and kept at 27 ± 1°C. All solutions were saturated with 95% O_2_/5% CO_2_.

After about 10 min of acclimation in the recording chamber, CRH neurons were recorded within 1 h. Infrared-differential interference contrast optics was used to visualize the neurons, and whole-cell patch clamp recordings were performed to detect the firing rate of CRH neurons. The electrodes (3–5 MΩ) back filled with internal solution (in mM: 130 K-gluconate, 4 KCl, 10 HEPES, 0.3 EGTA, 10 phosphocreatine-Na_2_, 4 Mg-ATP, 0.3 Na_2_-GTP) were used. Data were obtained by Clampex 11.1 (Molecular Devices, Inc., USA), filtered at 2 kHz, digitized at 10 kHz. Series resistances (10–25 MΩ) were monitored and any neurons that changed more than 20% during recording were discarded. Membrane potentials were held at –80 mV during the current clamp. In addition, to record the spontaneous firing in CRH neurons, the pipettes (3–5 MΩ) were back filled with ACSF, and cell-attached recordings were performed according to the procedures [21]. The holding current was kept at 0 pA by adjusting the holding potential during voltage clamp.

### 2.4 Statistical analysis

Data were presented as mean ± SEM. All analyses were performed by GraphPad Prism 9 (GraphPad Software, San Diego, CA, USA) and statistical significance was set at *p* < 0.05. Statistical significance was assessed using two-tailed Student’s *t*-tests or two-way repeated-measures analysis of variance (ANOVA) followed by Bonferroni *post*-*hoc* tests when appropriate.


**Ethical approval:** The research related to animals’ use has been complied with all the relevant national regulations and institutional policies for the care and use of animals. All procedure were approved by the Animal Advisory Committee at Zhejiang University.

## 3 Results

### 3.1 Cocaine exposure inhibits the firing rate of CRH neurons in the vBNST

**Figure 1:**
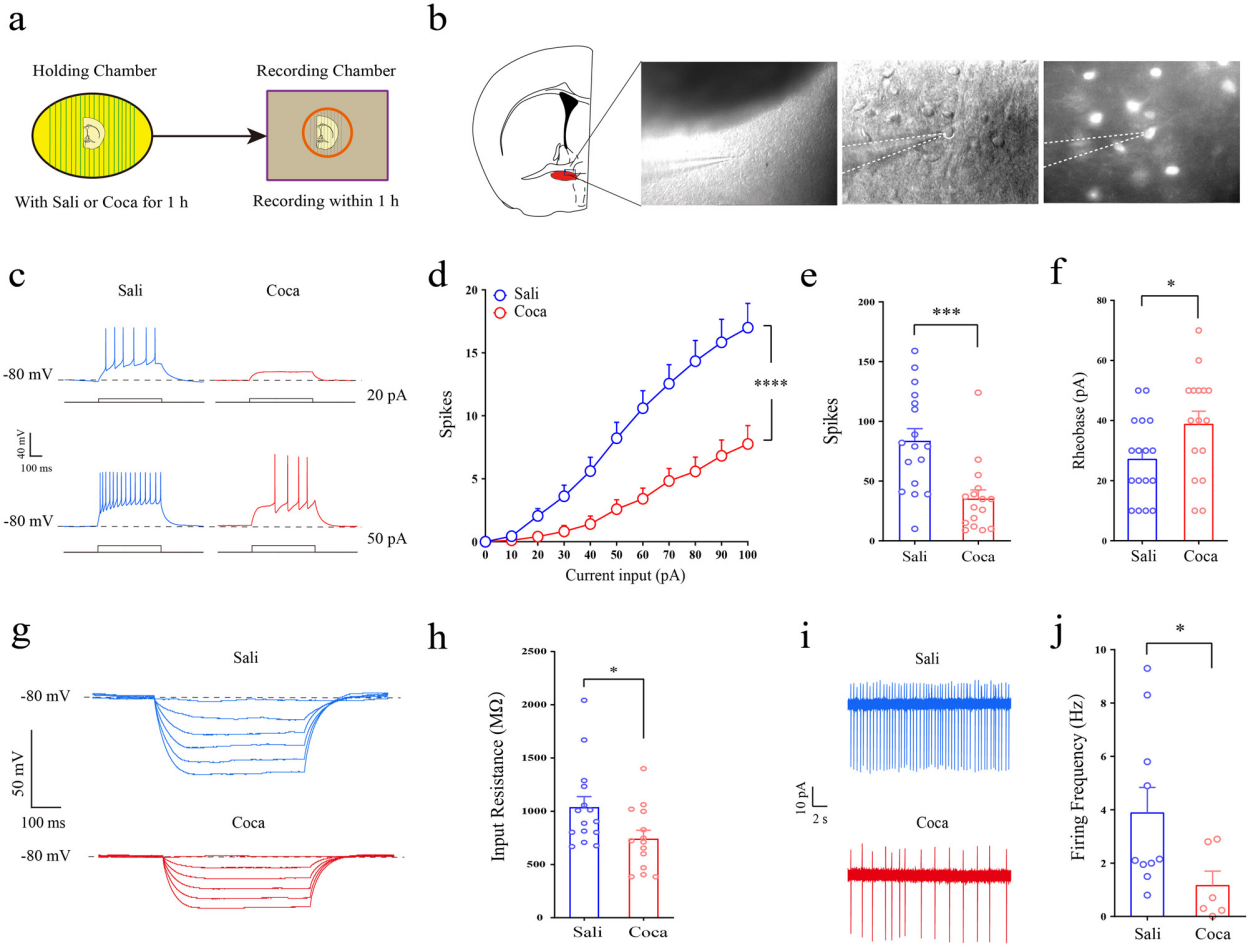
Cocaine exposure inhibits the firing rate of CRH neurons in the vBNST. (a) Schematic of the experimental procedure. (b) Sample graph of the patch clamp on fluorescent CRH neurons in the vBNST. (c) Samples of the firing rate induced by step current of 20 pA (above) and 50 pA (below) pretreated with saline or cocaine for 1 h. (d) Results of step current input and spikes, 500 ms duration at 1 Hz, 0 to +100 pA range with a 10 pA step increment after saline (*n* = 18 neurons/5 mice) or cocaine (*n* = 17 neurons/5 mice) treatment. Data were analyzed using two-way repeated-measures ANOVA and *post hoc* Bonferroni test; *** *p* < 0.001 vs Sali. (e) Firing rate comparison of all the steps of current injection. (f) Comparison of rheobase after saline (*n* = 18 neurons/5 mice) or cocaine (*n* = 17 neurons/5 mice) treatment. (g) Sample graph of the input resistance. (h) Statistical comparison of input resistance after saline (*n* = 18 neurons/5 mice) or cocaine (*n* = 17 neurons/5 mice) treatment. (i) Sample graph of the spontaneous firing rates. (j) Statistical comparison of spontaneous firing frequency after saline (*n* = 10 neurons/5 mice) or cocaine (*n* = 6 neurons/3 mice) treatment. Data were analyzed using two-tailed Student’s *t*-tests; *** *p* < 0.001 vs Sali, * *p* < 0.05 vs Sali.

To identify the effects of cocaine exposure on the intrinsic excitability of CRH neurons, we used 3 μM cocaine (corresponding to the concentration of cocaine *in vivo* when administered chronically at standard doses [10–20 mg/kg] [22]) to treat the brain slices containing vBNST, and incubated for 1 h before whole-cell recording. The patch clamp recording was done within 1 h after transferring the individual brain slices into the recording chamber (Figure 1a and b). The recording ACSF was mixed with 2 mM kynurenic acid and 100 μM picrotoxin to inhibit glutamate receptors and GABA_A_ receptors, respectively. During whole-cell recording, step currents (10 pA/step) were performed by the amplifier under current clamp, and the membrane potential was held at −80 mV. Our results revealed that cocaine exposure could dramatically decrease the firing rate of CRH neurons (Figure 1c and d; *F* = 13.40, df = 10, *p* < 0.0001). We also compared the total firing rate of all the steps of current injection, and showed that the firing rate decreased significantly after cocaine exposure (Figure 1e; *p* = 0.0003). Moreover, the rheobase was also increased after cocaine exposure (Figure 1f; *p* = 0.04), suggesting the reduction of excitability in CRH neurons. Accordingly, the membrane input resistance was attenuated after cocaine treatment (Figure 1g and h; *p* = 0.04), indicating the possibility of trafficking potassium channels onto the plasma membrane. In addition, we conducted cell-attached patch, and found that the spontaneous firing rate of CRH neurons was also depressed upon cocaine exposure (Figure 1i and j; *p* = 0.02).

### 3.2 Cocaine has no effects on the shape of action potential (AP)

**Figure 2:**
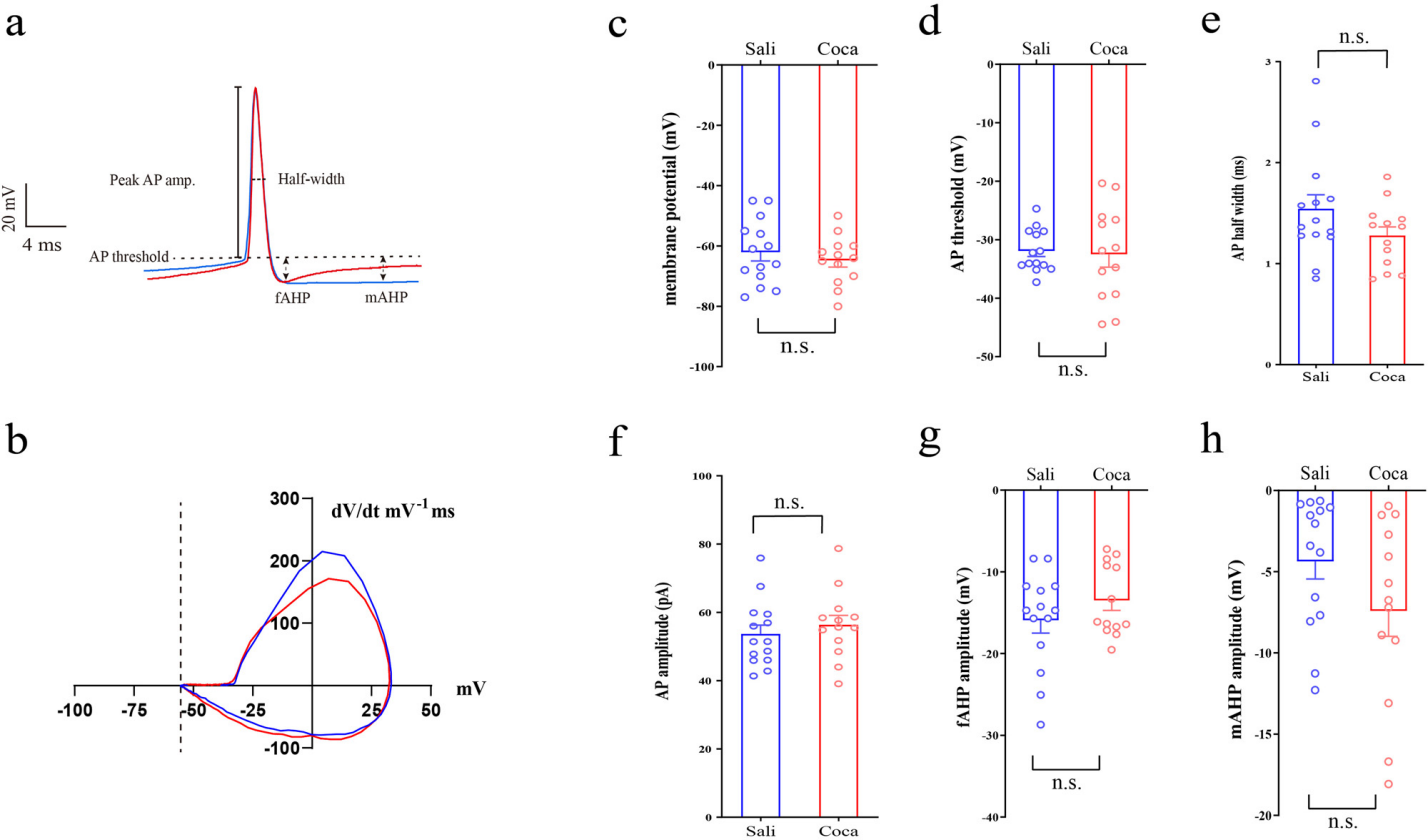
Cocaine has no effects on the shape of AP in CRH neurons. (a) Samples of AP of the first spikes (color blue represents Sali, red for Coca). (b) Sample phase-plane plots of AP for the first spikes (color blue represents Sali, red for Coca). Statistical results of (c) membrane potential, (d) AP threshold, (e) AP halfwidth, (f) AP amplitude, (g) fAHP, and (h) mAHP. Data were analyzed using two-tailed Student’s *t*-tests. Saline (*n* = 14 neurons/4 mice), cocaine (*n* = 13 neurons/5 mice). n.s. *p* > 0.05 vs Sali.

We analyzed the properties of the AP as well (Figure 2). The AP was induced by step currents (500 ms duration at 1 Hz, 0 to +100 pA range with a 10 pA step increment). All the AP recorded from each neuron were analyzed, and the data of properties were averaged. Compared with the saline treated ones, cocaine exposure has no effects on membrane potential (Figure 2c; *p* = 0.47), AP threshold (Figure 2d; *p* = 0.81), AP half-width (Figure 2e; *p* = 0.13), AP amplitude (Figure 2f; *p* = 0.49), fast after-hyperpolarization (fAHP) amplitude (Figure 2g; *p* = 0.68), medium after-hyperpolarization (mAHP) amplitude (Figure 2h; *p* = 0.12).

### 3.3 Sig-1Rs antagonist blocks the effects of cocaine on CRH neurons in the vBNST

**Figure 3:**
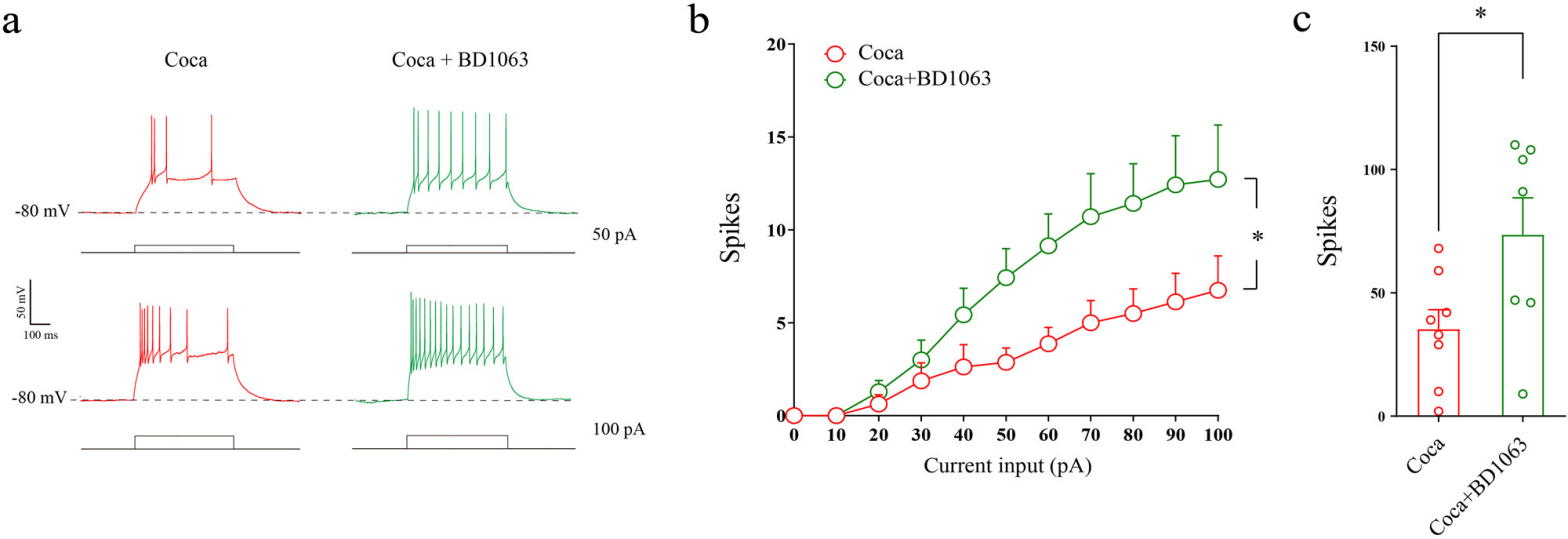
Sig-1Rs antagonist rescues the firing rate depression caused by cocaine exposure. (a) Samples of the firing rate induced by step current of 50 pA (above) and 100 pA (below) pretreated with cocaine or BD1063 plus cocaine for 1 h. (b) Results of step current input and spikes, 500 ms duration at 1 Hz, 0 to +100 pA range with a 10 pA step increment. Data were analyzed using two-way repeated-measures ANOVA and *post hoc* Bonferroni test (c) Firing rate comparison of all the steps of current injection. Data were analyzed using two-tailed Student’s *t*-tests. Cocaine (*n* = 8 neurons/5 mice), cocaine + BD1063 (*n* = 7 neurons/5 mice). * *p* < 0.05 vs Coca.

Given that the effects of cocaine on CRH neurons could act through Sig-1Rs, we pretreated the brain slices with a type of Sig-1Rs antagonist BD1063 (500 nM) for 30 min, then cocaine was added and incubated for 1 h before whole-cell recording. The patch clamp recording was conducted within 1 h after transferring the individual brain slices into the recording chamber as previously. The recording ACSF was also mixed with 2 mM kynurenic acid and 100 μM picrotoxin to inhibit glutamate receptors and GABA_A_ receptors, respectively. Our results showed that BD1063 could counteract the effect of cocaine on the firing rate of CRH neurons (Figure 3a and b; *F* = 5.54, df = 1, *p* = 0.04). We compared the total firing rate of all the steps as well, and found that BD1063 could significantly ameliorate the firing rate depression induced by cocaine exposure (Figure 3c; *p* = 0.04).

**Figure 4:**
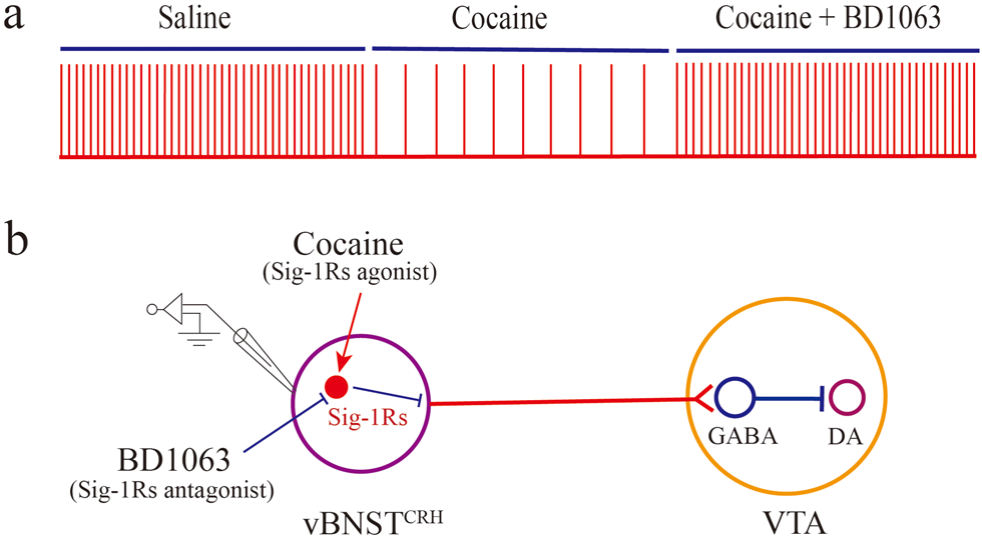
Schematic diagram of our experiment and the putative role of CRH neurons projecting to VTA. (a) Effects of cocaine and prototypical Sig-1Rs antagonist BD1063 on the firing rate of CRH neurons in the ventral BNST. (b) Effects of cocaine and BD1063 on Sig-1Rs, as well as the putative role in VTA. We propose that cocaine attenuates the intrinsic excitability of CRH neurons in the ventral BNST via Sig-1Rs. CRH neurons in the vBNST (vBNST^CRH^) projecting to GABA neurons in the VTA, while inhibiting the dopaminergic neurons. Thus, the firing rate depression of CRH neurons in the vBNST can lead to the activation of dopaminergic neurons in the VTA indirectly.

## 4 Discussion

Our investigations demonstrate that cocaine exposure causes firing rate depression in CRH neurons of the vBNST. Moreover, the input resistance of CRH neurons is also decreased, which may be associated with the augmentation of potassium channels in the plasma membrane. However, cocaine exposure has no effects on the shape of AP. Furthermore, we use BD1063 (Sig-1Rs antagonist) to treat the brain slices before cocaine exposure, and find that BD1063 can significantly inhibit the effects of cocaine on CRH neurons (Figure 4).

The comorbidity of addiction and anxiety suggests common underlying pathological neural circuits, and the vBNST CRH neurons should be critical ones [23]. It is noteworthy that BNST is implicated in drug seeking and relapse, typical symptoms of drug addiction. Series of articles demonstrate that drug addiction is associated with the excitability of neurons in the vBNST, such as alcohol-induced conditioned place preference (CPP) [24]. Besides, the vBNST neurons projecting to the VTA implicate in the expression CPP [5]. Interestingly, stressors exposure, such as predator odor or the elevated plus maze activates CRH neurons in the vBNST, yet non-threatening noxious odor inhibits CRH neurons in the vBNST [7]. Indeed, compelling evidence argue that the activation of stress response system, which is related to the excitability of vBNST CRH neurons contributes to compulsive drug seeking [25]. Therefore, the excitability of CRH neurons in the vBNST are crucial for cocaine addiction, specifically stress-induced drug craving and relapse. Our results indicate that cocaine exposure can inhibit the firing rate of CRH neurons, and thus may exert effects on anxiety and addiction. Notably, cocaine shows no obvious influence on the shape of AP, which in contrast is less important in coding functional or dysfunctional information in neurons [26]. However, the neuronal firing pattern and shape are subtly correlated, whereas the firing shape should in turn be more principal for presynaptic terminals [26]. Overall, our results may provide insight into the neural circuitry mechanisms contributing to the comorbidity of addiction and anxiety. Furthermore, based on the studies related to the mechanisms contributing to the alteration of intrinsic excitability in neurons, we propose Sig-1Rs as potential effectors underlying the firing rate depression in vBNST CRH neurons after cocaine exposure.

Cocaine addiction is considered as a type of pathological learning and memory, thus the alteration of neural plasticity underpinning such detrimental procedure. Indeed, cocaine addiction investigations traditionally focus on neurotransmitters, such as dopamine. However, the emerging role of Sig-1Rs played in cocaine addiction, highlights the “inside-out” effects of cocaine [16]. Residing at the endoplasmic reticulum (ER)–mitochondrion interface (mitochondrion-associated ER membrane, MAM), Sig-1Rs are a type of chaperone modulating Ca^2+^ signaling [27]. However, upon stimulation Sig-1Rs dynamically translocate to other regions of the neurons, regulating a range of ion channels [28]. Sig-1Rs are extensively distributed in the brain, especially the brain regions encoding motor functions, sensory, memory, as well as emotion [29–31]. In addition, accumulating evidence demonstrate that Sig-1Rs are of paramount important in drug addiction [32,33]. Remarkably, cocaine can bind to the Sig-1Rs and act as a type of agonist [14]. In turn, Sig-1Rs can modulate voltage-gated and ligand-gated ion channels, impacting the neuronal excitability and plasticity [34]. Previous studies showed that Sig-1Rs could mediate voltage-gated K^+^ channels bidirectionally [13]. More specifically, after binding with cocaine, Sig-1Rs subsequently act on Kv1.2 potassium channels, resulting in plasma membrane trafficking of Kv1.2 potassium channels [17,18]. Based on this, cocaine affects the intrinsic excitability of neurons, causing firing rate depression in the medium spiny neurons of NAc, which corresponds to our findings in vBNST CRH neurons [35]. Furthermore, Sig-1Rs also regulate the synaptic plasticity of neurons [36] and may therefore exert its effects on learning and memory [37], yet such effects are beyond the scope of our current investigations.

It is well-known that cocaine acts as a dopamine transporter (DAT) inhibitor, and has a high-binding affinity to DAT (0.2–0.6 μM) [38]. However, the dopaminergic projections primarily target dorsal BNST. Moreover, cocaine may inhibit CRH neurons via blocking voltage-gated sodium channels. But cocaine needs no less than 30 μM to block voltage-gated sodium channels effectively [39]. Considering the low concentration of cocaine (3 μM) used in our research, cocaine has little effect on sodium currents. In contrast, the affinity of cocaine for Sig-1Rs is about 2 μM, which is within the range of concentration used in our study.

Finally, it should be clarified that the lack of data from *in vivo* exposure of cocaine is the limitation of our investigation, and it is valuable to validate the relevance of the *in vitro* slice studies. We will consider the effects of single dose of cocaine on the firing rate of CRH neurons *in vivo* in our future studies. Furthermore, our investigation only focuses on CRH neurons in the vBNST and have not examined the excitability of non-CRH neurons in the vBNST. Thus, we have no idea if vBNST CRH neurons are uniquely susceptible to the actions of cocaine or would a similar effect be observed in any neuron examined.

Altogether, our investigation reveals the effects of cocaine on the intrinsic excitability of CRH neurons in the vBNST, and suggests Sig-1Rs as the targets of cocaine intracellularly (Figure 4). Given the essential role of vBNST CRH neurons in stress and anxiety, further research are needed to illustrate the effects of Sig-1Rs in the CRH neurons on these psychiatric diseases more directly, such as what is the effect of knock out Sig-1Rs in the vBNST CRH neurons on anxiety as well as for the process of cocaine addiction. Expecting mounting investigations about the mechanisms of Sig-1Rs would be useful in bridging the gap between academic research and regarding Sig-1Rs as therapeutic targets in treating neuropsychiatric diseases.
